# Developmental Programming of PCOS Traits: Insights from the Sheep

**DOI:** 10.3390/medsci7070079

**Published:** 2019-07-11

**Authors:** Rodolfo C. Cardoso, Vasantha Padmanabhan

**Affiliations:** 1 Department of Animal Science, Texas A&M University; 2471 TAMU, College Station, TX 77843-2471, USA; 2 Department of Pediatrics; University of Michigan; 7510 MSRB I, Ann Arbor, MI 48109-5718, USA

**Keywords:** PCOS, sheep, testosterone, interventions

## Abstract

Polycystic ovary syndrome (PCOS) is a complex disorder that results from a combination of multiple factors, including genetic, epigenetic, and environmental influences. Evidence from clinical and preclinical studies indicates that elevated intrauterine androgen levels increase the susceptibility of the female offspring to develop the PCOS phenotype. Additionally, early postnatal endocrine and metabolic imbalances may act as a “second-hit”, which, through activational effects, might unmask or amplify the modifications programmed prenatally, thus culminating in the development of adult disease. Animal models provide unparalleled resources to investigate the effects of prenatal exposure to androgen excess and to elucidate the etiology and progression of disease conditions associated with this occurrence, such as PCOS. In sheep, prenatal treatment with testosterone disrupts the developmental trajectory of the fetus, culminating in adult neuroendocrine, ovarian, and metabolic perturbations that closely resemble those seen in women with PCOS. Our longitudinal studies clearly demonstrate that prenatal exposure to testosterone excess affects both the reproductive and the metabolic systems, leading to a self-perpetuating cycle with defects in one system having an impact on the other. These observations in the sheep suggest that intervention strategies targeting multiple organ systems may be required to prevent the progression of developmentally programmed disorders.

## 1. Introduction

Globally, approximately 60–80 million people experience difficulty conceiving [1], and, in 30–40% of couples of childbearing age seeking fertility counseling, infertility is exclusively a problem with the female. Among the infertility disorders, polycystic ovary syndrome (PCOS) is one of the most common, affecting approximately five million women in the USA and over 100 million globally [2]. PCOS is characterized by reproductive manifestations that may include oligo-/anovulation, polycystic ovarian morphology, luteinizing hormone (LH) hypersecretion, and hyperandrogenism [3]. In addition, approximately 70% of PCOS patients exhibit metabolic disturbances, such as obesity and insulin resistance [4]. Despite the high prevalence of PCOS, a clear understanding of the etiology and progression of this syndrome remains elusive.

A wealth of research in clinical cohorts and animal models indicate that PCOS is a complex disorder that results from a combination of multiple factors, including genetic, epigenetic, and environmental influences. While recent genome-wide association studies (GWAS) identified several susceptibility loci in PCOS patients, the heritability currently accounted for by the known loci is less than 10% [5,6]. Although additional loci, specific genes and functional variants of interest are likely to be identified by GWAS and other genetic approaches, the limited heritability accounted for by these studies so far suggests that other factors such as epigenetics and in utero environmental insults may play a role. The most widely implicated environmental insult associated with PCOS is the perinatal (prenatal and early postnatal) exposure to high levels of androgens. Evidence from clinical and preclinical studies indicates that elevated intrauterine androgen levels increase the susceptibility of the female offspring to develop the PCOS phenotype [7,8]. Women with congenital adrenal hyperplasia, a condition that results in abnormally high prenatal androgen exposure, have a considerably greater likelihood of developing the PCOS reproductive phenotype [9]. Moreover, recent studies [10,11,12] point to a higher prevalence of reproductive and metabolic dysfunction in the offspring of women with PCOS, who also manifest hyperandrogenism during pregnancy [13].

This notion that the adult phenotype can be shaped during fetal life is supported by the “developmental origins of health and disease (DOHaD)” hypothesis by Barker and colleagues [14] and has gained considerable momentum after the emergence of epidemiological data from the 1944, 1945 Dutch famine cohort. These data demonstrated that maternal malnutrition during gestation is associated with a marked increase in the risks of the offspring for developing cardiovascular and metabolic diseases [15]. These findings, in conjunction with subsequent clinical and animal studies [16,17,18,19], unequivocally demonstrate that the perinatal period, a period in which organogenesis and tissue differentiation occur through a tightly controlled and timed process, is a critical window of opportunity for programming the offspring’s phenotype.

**Figure 1:**
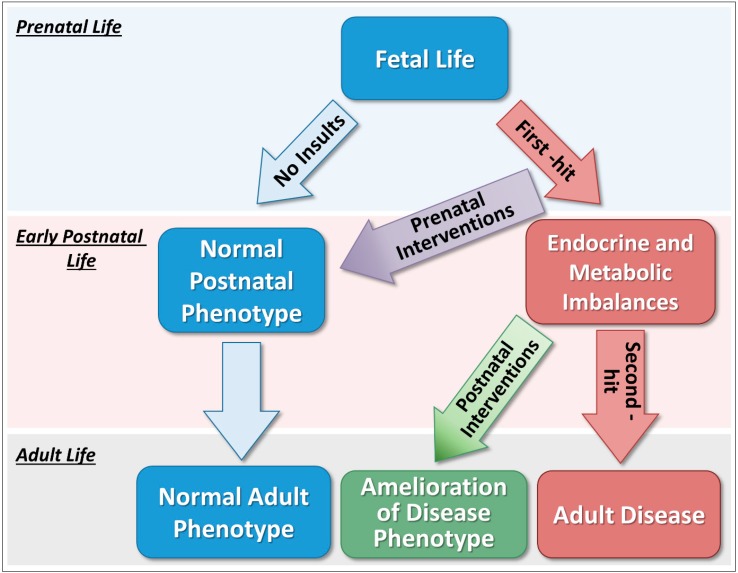
Two-hit hypothesis for adult onset of diseases. Insults occurring during the prenatal life constitute a “first-hit” that can lead to reorganization of several organ systems. Despite these modifications, this “first-hit” alone might be insufficient to alter the adult phenotype resulting in disease. However, endocrine and metabolic imbalances occurring later in life may act as a “second-hit”, which through activational effects might unmask or amplify the modifications programmed prenatally, thus culminating in the development of adult disease. On the other hand, prenatal interventions that can negate the effects of “first-hit” insults may successfully prevent the manifestation of some disease traits. Postnatal interventions that avert the effects of “second-hit” stressors may ameliorate the disease phenotype by preventing the manifestation of some disease traits.

In addition to the well-characterized impact during fetal development, recent research observations support a “two-hit” hypothesis to explain the onset as well as severity of diseases [20,21]. This hypothesis proposes that an insult occurring during the prenatal life constitutes a “first-hit” that combined with genetic susceptibility can lead to reorganization of several organ systems. Despite these modifications during early life, in many occasions this “first-hit” alone might be insufficient to alter the adult phenotype resulting in disease. However, endocrine and metabolic imbalances occurring later in life and/or exposure to adverse stressors may act as a “second-hit”, which through activational effects might unmask or amplify the modifications programmed prenatally, thus culminating in the development of adult disease [21]. On the other hand, postnatal interventions that avert the effects of “second-hit” stressors may successfully prevent the manifestation or reduce the severity of some disease traits despite the programming effects of prenatal insults (Figure 1). While several mechanisms may be involved in this process, mounting evidence suggests that epigenetic mechanisms, such as DNA methylation, histone modification and non-coding RNAs, mediate the effects of endogenous or exogenous factors on the developmental plasticity of specific organ systems [20]. The “two-hit” hypothesis is supported by our observations in the sheep model of PCOS, in which many reproductive disease traits programmed by prenatal testosterone excess can be prevented from manifesting themselves if postnatal endocrine and metabolic imbalances are managed properly. Focusing primarily on findings from the sheep model of PCOS, this review summarizes the effects of prenatal and postnatal interventions on preventing or mitigating the adverse effects of prenatal testosterone excess on reproductive and metabolic function.

## 2. Sheep Model of Polycystic Ovary Syndrome Phenotype

Animal models provide unparalleled resources to investigate the effects of perinatal exposure to androgen excess and to elucidate the etiology and progression of disease conditions associated with this occurrence, such as PCOS. Most studies have used rodents, sheep, and non-human primates as research models and comparative aspects of these different animal models have been discussed previously [22,23]. Our research group has carried out multiple longitudinal studies in the female sheep to carefully characterize the phenotype of these animals at multiple developmental time points. This is one of the few animal models of PCOS phenotype in which an extensive longitudinal characterization has been performed. There are numerous benefits of using the female sheep for endocrine and reproductive research. Sheep are not litter bearing and are amenable to different surgical and experimental procedures and interventions; their large size allows for detailed and repetitive hormonal profiling and in vivo sampling and measurement of hypothalamic neuropeptides. Moreover, since sheep are domesticated, they are kept in a natural environment and not subject to the stress effects associated with caging. Additionally, because sheep is a precocial species, the trajectory of development of several organs systems, such as the ovary and pancreas, follows a similar pattern as that of humans [23], thus having valuable translational relevance. 

The normal gestation period of ewes is approximately 147 days, ranging from 142 to 155 days. In our experiments investigating the effect of prenatal androgen excess on the offspring, we have used different experimental paradigms to gain insight into the susceptibility windows and mechanisms involved in this process. A comparison of female sheep treated with testosterone from gestational day (GD) 30–90 vs. GD 60–90 has identified critical periods in which specific reproductive and metabolic disorders are programmed. Moreover, a comparison of the effects of prenatal treatment with either testosterone, dihydrotestosterone (DHT; a non-aromatizable androgen), or co-treatment with testosterone and the androgen antagonist flutamide has identified specific signaling mechanisms responsible for programming disease traits in this sheep model. This review will focus primarily on phenotypic traits from the GD 30–90 model that has been studied more extensively. For comprehensive reviews comparing the different experimental paradigms and the resulting phenotypes, as well as tissue-specific findings associated with reproductive and metabolic alterations in this sheep model, readers are referred to Cardoso, et al. [24], and Padmanabhan and Veiga-Lopez [23]. 

Prenatal treatment with testosterone disrupts the developmental trajectory of the ovine fetus culminating in adult neuroendocrine, ovarian, and metabolic perturbations that closely resemble those seen in women with PCOS [23]. Prenatal testosterone treatment from GD 30–90 compromises reproductive function, resulting in the progressive deterioration of ovarian cyclicity, compromised fertility, and premature reproductive failure, with most females becoming anovulatory by the second breeding season (early adulthood) [25]. Because the use of sheep allows detailed hormonal profiling, our studies indicate that the progressive reproductive failure seen in prenatal testosterone-treated females stems, at least in part, from tonic activation of the reproductive neuroendocrine axis. Prenatal testosterone-treated sheep present defects in all three steroid feedback mechanisms controlling gonadotropin-releasing hormone (GnRH) and LH secretion, namely estradiol negative [26], estradiol positive [27], and progesterone negative feedback [28,29]. Furthermore, pituitary sensitivity to GnRH is markedly increased in these animals [30]. The defects in steroid negative feedback and augmented pituitary responsiveness to GnRH together contribute to the LH excess and consequent functional hyperandrogenism seen in prenatal testosterone-treated sheep.

In addition to reproductive neuroendocrine disruptions, prenatal treatment with testosterone results in a polyfollicular ovary [31,32]. This polyfollicular phenotype likely stems from an abnormal increase in follicular recruitment associated with arrest in antral follicular development causing persistence [33]. This premise is supported by observations that prenatal testosterone results in lower percentage of primordial follicles and higher percentage of primary and secondary follicles in the ovarian cortex of adult sheep, suggesting increased follicular recruitment [34]. Serial ultrasonographic studies demonstrated that in addition to the increased follicular recruitment, prenatal testosterone treatment results in several antral follicles that survive for longer periods, thus indicating follicular persistence [35]. Interestingly, the polyfollicular ovarian morphology and increased follicular persistence seen in sheep prenatally treated with testosterone were not evident in females treated with DHT, suggesting that the aromatization of testosterone into estradiol is necessary to program these ovarian perturbations [32,36].

From a cardio-metabolic standpoint, prenatal treatment with testosterone leads to insulin resistance and compensatory hyperinsulinemia [37], altered visceral adiposity and adipocyte size [38], impaired adipocyte differentiation [39], and hypertension [40]. Our detailed longitudinal studies characterizing glucose–insulin homeostasis in this PCOS sheep model have identified significant fluctuations in insulin sensitivity throughout life [41]. During infantile [37] and early juvenile development [42], ewes prenatally exposed to testosterone excess exhibit a significant reduction in insulin sensitivity. Conversely, during peripubertal life, prenatal testosterone-treated sheep demonstrate marked improvements in insulin sensitivity, exhibiting greater insulin sensitivity index than control females during an euglycemic-hyperinsulinemic clamp [38]. However, at later adult life, prenatal treatment with testosterone results in the reestablishment of insulin resistance [42]. In conjunction, these longitudinal studies suggest that a period of compensatory adaptation of metabolic tissues to prenatal exposure to testosterone excess occurs around pubertal development in sheep [41]. 

At the adipose tissue level, prenatal treatment with testosterone reduced visceral adiposity and increased the ratio of small to large adipocytes in the visceral and subcutaneous adipose compartments [38,41]. While it was originally proposed that hypertrophy of adipocytes was linked to insulin resistance [43,44], recent studies investigating adipocyte size in obese but otherwise healthy patients have shown that the small to large adipocyte ratio is actually higher in insulin-resistant compared to insulin-sensitive patients [45,46]. Thus, it is believed that insulin resistance in some obese patients may originate from failure of a subset of smaller adipocytes to fully differentiate into mature adipocytes with increased capacity to store lipids [47]. Consequently, excess free fatty acids are accumulated in other metabolic tissues such as liver and muscle, leading to lipotoxicity, oxidative stress, and subsequent insulin resistance [48,49,50]. In agreement with these findings, prenatal treatment with testosterone not only resulted in reduced visceral adiposity and smaller adipocytes in sheep [38,41], but it also resulted in ectopic lipid accumulation in the liver and skeletal muscle [51], and elevated concentrations of total and saturated free fatty acids [38]. Thus, these observations suggest that reduced visceral adiposity and increased ratio of small to large adipocytes may be the earliest events in the development of metabolic dysfunctions, such as dyslipidemia and insulin resistance, in this sheep model. Alternatively, an increased proportion of small adipocytes may represent a compensatory mechanism that develops only after insulin resistance is established as an attempt to maintain glucose–insulin homeostasis. In a recent study, normal-weight women with PCOS exhibited a significant increase in the percentage of small subcutaneous adipocytes when compared with the control group [52], similar to observed in the sheep model of PCOS phenotype. In that study, authors propose that the greater proportion of small subcutaneous adipocytes in normal-weight women with PCOS likely represents enhanced adipocyte hyperplasia in an attempt to increase lipid storage and improve adipose insulin sensitivity [52].

In addition to metabolic perturbations, prenatal exposure to testosterone excess leads to the programming of cardiovascular dysfunction in the female offspring [53]. Prenatal testosterone treatment results in hypertension and a tendency for a higher heart rate compared to control sheep [40]. At the cardiac level, prenatal exposure to testosterone excess increased the expression of several molecular markers involved in insulin signaling and associated with cardiac hypertrophy [54]. Moreover, histological investigations reported myocardial disarray and increased cardiomyocyte diameter in these animals [54]. Collectively, these observations suggest that prenatal testosterone excess results in adverse left ventricular remodeling, which likely contributes to the development of adult hypertension in sheep. 

To understand the pathways and mechanisms linking prenatal testosterone excess with adult disease traits, it is critical to identify the impacts on the fetomaternal endocrine and metabolic milieus. In sheep, prenatal treatment with testosterone increases not only the maternal but also the fetal concentrations of androgens and estradiol [55,56], suggesting that the programming of adult disease could occur via both androgenic and estrogenic pathways. Gestational testosterone excess also reduces maternal concentrations of progesterone, increases circulating insulin levels, and disrupts the maternal–fetal correlations for several metabolites [56]. Together, these findings demonstrate that gestational testosterone treatment disrupts the fetomaternal steroidal and metabolic milieus, which are likely key modifications programming disease traits in this sheep model. 

To dissect out the contribution of each pathway, we have performed a series of experiments investigating the effects of prenatal and postnatal treatment with an androgen antagonist or an insulin sensitizer on the reproductive and metabolic phenotypes of female sheep prenatally treated with testosterone. We focused primarily on the actions of androgens and insulin since these hormones play an important role in modulating the development of several organ systems [19] and are markedly altered due to gestational testosterone treatment. Below, we summarize the main effects of prenatal and postnatal interventions in preventing or ameliorating some of the disease traits in this sheep model. These effects are also summarized in Table 1. 

## 3. Effects of Prenatal Interventions on Reproductive and Metabolic Phenotypes

Prenatal co-treatment with testosterone and flutamide, an androgen antagonist, provides valuable insights into the role of the androgenic pathway in programming adult disease traits. Importantly, the co-treatment with flutamide has been shown to effectively block the effects of endogenous and exogenous androgens on phenotypic virilization in males and prenatal testosterone treated female sheep, respectively [61]. Prenatal co-treatment with flutamide has been shown to prevent the advancement in pubertal onset induced by prenatal treatment with testosterone [57], suggesting that androgen signaling is involved in programming this perturbation. This is supported by observations that prenatal DHT treatment also leads to advanced neuroendocrine puberty in the female offspring [58]. Moreover, prenatal co-treatment with flutamide successfully prevented the reduction in estradiol negative feedback seen in prenatal testosterone-treated ewes [59], suggesting that this neuroendocrine defect is also programmed via androgenic actions of testosterone. On the other hand, disruptions in estradiol positive feedback were observed in testosterone- but not DHT-treated sheep suggesting that this alteration is programmed via estrogenic actions of prenatal testosterone [58,59]. The findings that prenatal co-treatment with flutamide failed to reverse the defects in estradiol positive feedback support this premise [51]. 

Prenatal co-treatment with flutamide was also shown to restore estrous synchronization response and partially improve LH surge release after prostaglandin injection in prenatal testosterone-treated females [57]. This observation supports the premise that activation of the androgen receptor is also involved in programming LH preovulatory surge defects in this sheep model. Therefore, these findings do not support the aforementioned notion that LH surge defects in prenatal testosterone-treated sheep are programmed only by estrogenic actions of testosterone due to aromatization into estradiol. These observations in female sheep prenatally co-treated with testosterone and flutamide raise the possibility that the effects of DHT may not be mediated exclusively via androgenic pathways but rather due to conversion of DHT into 3β-androstenediol and acting through estrogen-β receptors [62]. Alternatively, both androgens and estrogens may have synergistic effects organizing the neuroendocrine components responsible for the LH preovulatory surge. This is supported by the observation that despite all females prenatally co-treated with flutamide exhibited LH surges, the LH surges were not of comparable magnitude with those observed in control females [57]. 

While some of the neuroendocrine defects were successfully prevented by co-treatment with flutamide, metabolic alterations were not prevented by prenatal co-treatment with flutamide. Flutamide failed to prevent insulin resistance [41], alterations in adipocyte morphology [41], and oxidative stress [51] in prenatal testosterone-treated sheep. Collectively, these findings suggest that the androgenic pathway plays a key role in programming neuroendocrine and reproductive defects but is likely less critical in programming metabolic perturbations in this sheep model of PCOS phenotype. Therefore, other signaling pathways, such as activation of estrogen and/or insulin receptors, are potential mechanisms by which prenatal testosterone excess can lead to metabolic dysfunction in the female offspring. 

Because gestational testosterone treatment results in maternal hyperinsulinemia [56], it is plausible that some of the adult abnormalities programmed in the offspring are mediated via the hyperactivation of the insulin signaling pathway. Therefore, we have performed multiple experiments investigating the effects of prenatal co-treatment with testosterone and rosiglitazone, an insulin sensitizer. Importantly, the dose of rosiglitazone used in these studies (8 mg/day) is within the dose range used to treat PCOS women [63,64], and has been shown to restore insulin sensitivity in insulin-resistant sheep [65]. Similar to prenatal co-treatment with an androgen antagonist, prenatal co-treatment with rosiglitazone prevented the high incidence of precocious puberty seen in prenatal testosterone-treated sheep [57]. However, this process was not associated with improvements in the neuroendocrine feedback mechanisms or in the restoration of normal preovulatory LH surge dynamics. Additionally, prenatal co-treatment with an insulin sensitizer failed to prevent the premature reproductive failure seen in prenatal testosterone-treated sheep [57]. Therefore, these observations corroborate the notion that the androgenic pathway is likely the central mechanism programming neuroendocrine and reproductive defects in this sheep model.

From a metabolic standpoint, prenatal co-treatment with an insulin sensitizer successfully prevented the development of insulin resistance in prenatal testosterone-treated sheep, restoring mean insulin and insulin/glucose ratio during a glucose tolerance test [41]. These observations suggest that gestational hyperinsulinemia plays a role in programming insulin resistance in the offspring. In support of this concept, studies in rodents demonstrate that other conditions resulting in gestational hyperinsulinemia such as maternal obesity [66] and protein restriction [67] during gestation are also associated with the development of insulin resistance in the offspring. While the exact mechanisms by which prenatal co-treatment with an insulin sensitizer improves metabolic function in sheep remain elusive, inhibition of a prenatal testosterone-induced rise in proinflammatory cytokines [51] and reduction in adipocyte differentiation [39] are likely involved. In summary, these collective observations suggest that gestational hyperinsulinemia alters the normal developmental trajectory of fetal metabolic tissues leading to the development of adult insulin resistance in the offspring.

## 4. Effects of Postnatal Interventions on Reproductive and Metabolic Phenotypes

While modifications in utero can result in the reorganization of several organ systems that are associated with later development of reproductive and metabolic impairments, it is now evident that endocrine and metabolic imbalances occurring postnatally may be required to unmask or amplify some disease traits [20,21]. Because prenatal testosterone-treated sheep manifest functional hyperandrogenism and insulin resistance associated with hyperinsulinemia postnatally, it is conceivable that these endocrine imbalances are required for the full manifestation of the PCOS phenotype. Therefore, based on these observations, we performed multiple studies investigating the effects of postnatal treatment with an androgen antagonist or an insulin sensitizer on the phenotype of these animals. 

Similar to the effects of prenatal interventions, postnatal treatment with the androgen antagonist flutamide starting at weaning (approximately eight weeks of age) prevented the advancement on pubertal maturation in this sheep model [57]. These findings suggest that while modifications occurring during fetal life are critical for programming precocious puberty in female sheep, elevated androgen action postnatally is likely also involved. This is consistent with the premise that environmental, endocrine, and metabolic cues during early postnatal life clearly play a role in controlling the timing of puberty in females [60,68,69]. In regards to the estradiol positive feedback and preovulatory LH surge, while postnatal treatment with an androgen antagonist failed to normalize the timing of the LH surge, it increased the total LH released in response to the estradiol positive feedback challenge to control levels [70]. This is supportive of increased androgen signaling underlying reduced LH surge in prenatal testosterone-treated females. It is possible that postnatal androgen antagonist treatment normalizes the total LH released during the surge by restoring normal androgen actions in the neuroendocrine axis, since prenatal testosterone treatment increases the expression of the androgen receptor in both the hypothalamus [71] and pituitary gland of adult sheep [30]. Similar findings were reported in women with PCOS, in which treatment with the androgen antagonist flutamide improved LH pulse frequency and restored ovulation [72,73]. 

As anticipated, postnatal treatment with the insulin sensitizer rosiglitazone improved peripheral insulin sensitivity and normalized insulin levels in prenatal testosterone-treated sheep [65]. However, the effects of postnatal treatment with rosiglitazone were not only limited to metabolic function, but also markedly improved several reproductive traits in this sheep model of PCOS phenotype. Insulin sensitizer treatment successfully prevented advancement of puberty in prenatal testosterone-treated sheep [57] and prevented the premature reproductive failure seen in these animals [65]. While 80% of prenatal testosterone-treated females showed a reduced number of estrous cycles in the second breeding season (early adulthood), only 20% of females postnatally treated with rosiglitazone showed such deterioration [65]. Postnatal treatment with rosiglitazone also decreased the number of aberrant cycles (≥18 days) during the second breeding season in comparison with prenatal testosterone-treated females that did not receive insulin sensitizer [65]. 

These positive effects on reproductive function are associated with improved neuroendocrine function. Postnatal insulin sensitizer treatment partially improved the estradiol positive feedback by increasing the magnitude (total LH released in response to positive feedback challenge) as well as amplitude (difference between peak and nadir) of the LH surge [70]. These observations suggest an activational role for insulin in modulating the LH surge magnitude. Previous observations that insulin infusion dampens the magnitude of the LH surge in sheep [74] are consistent with the reduced LH surge magnitude seen in prenatal testosterone-treated sheep, which are hyperinsulinemic [42]. While the exact mechanisms by which rosiglitazone improves the preovulatory LH surge are unknown, they may involve restoration of LH releasable pool in the anterior pituitary and/or normalization of hypothalamic or pituitary responsiveness to the estradiol positive feedback. In addition to improved preovulatory LH surge, insulin sensitizer treatment also normalized LH tonic (pulsatile) release by normalizing the pituitary sensitivity to GnRH and preventing the pulsatile LH hypersecretion [30], thus suggesting that postnatal perturbations in insulin-glucose homeostasis contribute to this neuroendocrine perturbation. Because LH hypersecretion (tonic release) disrupts follicular development and steroidogenesis, it is likely that the beneficial effects of postnatal rosiglitazone treatment on reproduction are mediated, in part, at the pituitary gonadotrope level.

## 5. Conclusions 

In conclusion, epidemiological findings in humans and studies in prenatal testosterone-treated animal models, including the sheep, indicate that alterations programmed in utero play an important role in the development and manifestation of the PCOS phenotype during adulthood. However, the interactions between prenatal insults and the early postnatal environment in the development and manifestation of adult diseases remain poorly understood. Animal models of fetal programming serve as an important research tool to investigate these interactions. Our studies in the female sheep clearly demonstrate that prenatal exposure to testosterone excess affects both the reproductive and the metabolic systems, leading to a self-perpetuating cycle with defects in one system having an impact on the other [19]. Thus, intervention strategies targeted at multiple organ systems may be required to prevent the progression of some developmentally programmed disorders. This seems to be the case for PCOS patients, in which combined anti-androgen and insulin-sensitizing treatment has additive benefits on several metabolic traits when compared to monotherapies [75]. Studies in prenatal testosterone-treated sheep testing the combined anti-androgen and insulin-sensitizing treatment are warranted. Additionally, further investigation of potential common mediators affecting the different systems may help identify early biomarkers and therapeutic targets for preventive interventions. Nonetheless, since the long-term consequences of pharmacological interventions during the preconception and gestational periods remain unknown, preventive strategies should focus on promoting healthy lifestyle choices and minimizing exposure to potentially harmful agents.

## Figures and Tables

**Table 1:** Reproductive and metabolic disease traits in prenatal testosterone- and dihydrotestosterone (DHT)-treated sheep (2 to 3 years of age—early adulthood) and the effectiveness of prenatal or postnatal interventions with either an androgen antagonist or an insulin sensitizer.

Traits	Prenatal Testosterone	Prenatal DHT	Interventions—Pathology Manifestations			
			Prenatal Testosterone + Prenatal Androgen Antagonist	Prenatal Testosterone + Prenatal Insulin Sensitizer	Prenatal Testosterone + Postnatal Androgen Antagonist	Prenatal Testosterone + Postnatal Insulin Sensitizer
**Reproductive Traits**						
Advanced puberty	Yes [57]	Yes ^Ψ^ [58]	No [57]	No [57]	No [57]	No [57]
Functional Hyperandrogenism	Yes [23]	Yes [23]	Not tested	Not tested	Not tested	Not tested
PCO morphology	Yes [31]	No [31]	Not tested	Not tested	Not tested	Not tested
Disrupted preovulatory LH surge	Yes [57]	No [59]	Partially [57]	Yes [57]	Yes [57]	Yes [57]
Disrupted estradiol positive feedback	Yes [60]	No [58,59]	Yes [60]	Yes [60]	Partially [60]	Partially [60]
Disrupted estradiol negative feedback	Yes [26]	Yes [59]	No ^Σ^ [61]	Not tested	Not tested	Not tested
GnRH-stimulated LH hypersecretion	Yes [30]	Yes [30]	Yes [30]	Partially [30]	Yes [30]	No [30]
Increased follicular recruitment	Yes [33]	Yes [33]	Not tested	Not tested	Not tested	Not tested
Follicular persistence	Yes [35]	No [36]	Not tested	Not tested	Not tested	Not tested
**Metabolic Traits**						
Insulin resistance	Yes [38,41]	Yes [41]	Yes [41]	No [41]	Yes ^#^	No ^#^
Altered visceral adiposity	Yes [38]	Not tested	Not tested	Not tested	Not tested	Not tested
Altered adipocyte size	Yes [38,41]	Not tested	Partially [41]	Partially [41]	Not tested	Not tested
Adipocyte differentiation	Reduced [39]	Not tested	Partially [39]	Partially [39]	Not tested	Not tested
Hypertension	Yes [40]	Not tested	Not tested	Not tested	Not tested	Not tested

^Ψ^ Neuroendocrine puberty; ^#^ Unpublished observations: ^Σ^ Based on escape from estradiol negative feedback. PCO: polycystic ovary; LH: luteinizing hormone; GnRH: gonadotropin-releasing hormone.
